# Clusters of Drug-Resistant *Mycobacterium tuberculosis* Detected by Whole-Genome Sequence Analysis of Nationwide Sample, Thailand, 2014–2017

**DOI:** 10.3201/eid2703.204364

**Published:** 2021-03

**Authors:** Ditthawat Nonghanphithak, Angkana Chaiprasert, Saijai Smithtikarn, Phalin Kamolwat, Petchawan Pungrassami, Virasakdi Chongsuvivatwong, Surakameth Mahasirimongkol, Wipa Reechaipichitkul, Chaniya Leepiyasakulchai, Jody E. Phelan, David Blair, Taane G. Clark, Kiatichai Faksri

**Affiliations:** Khon Kaen University, Khon Kaen, Thailand (D. Nonghanphithak, W. Reechaipichitkul, K. Faksri);; Mahidol University, Bangkok, Thailand (A. Chaiprasert, C. Leepiyasakulchai);; Ministry of Public Health, Bangkok (S. Smithtikarn, P. Kamolwat, P. Pungrassami);; Prince of Songkla University, Hat Yai, Songkhla, Thailand (V. Chongsuvivatwong);; Ministry of Public Health, Nonthaburi, Thailand (S. Mahasirimongkol);; London School of Hygiene and Tropical Medicine, London, UK (J.E. Phelan, T.G. Clark);; James Cook University, Townsville, Queensland, Australia (D. Blair)

**Keywords:** *Mycobacterium tuberculosis*, drug resistance, multidrug-resistant TB, transmission, whole-genome sequencing, Thailand, bacteria, antimicrobial resistance, tuberculosis and other mycobacteria

## Abstract

Multidrug-resistant tuberculosis (MDR TB), pre-extensively drug-resistant tuberculosis (pre-XDR TB), and extensively drug-resistant tuberculosis (XDR TB) complicate disease control. We analyzed whole-genome sequence data for 579 phenotypically drug-resistant *M. tuberculosis* isolates (28% of available MDR/pre-XDR and all culturable XDR TB isolates collected in Thailand during 2014–2017). Most isolates were from lineage 2 (n = 482; 83.2%). Cluster analysis revealed that 281/579 isolates (48.5%) formed 89 clusters, including 205 MDR TB, 46 pre-XDR TB, 19 XDR TB, and 11 poly–drug-resistant TB isolates based on genotypic drug resistance. Members of most clusters had the same subset of drug resistance-associated mutations, supporting potential primary resistance in MDR TB (n = 176/205; 85.9%), pre-XDR TB (n = 29/46; 63.0%), and XDR TB (n = 14/19; 73.7%). Thirteen major clades were significantly associated with geography (p<0.001). Clusters of clonal origin contribute greatly to the high prevalence of drug-resistant TB in Thailand.

Tuberculosis (TB), caused by *Mycobacterium tuberculosis*, is a major global public health issue. Southeast Asia contributes notably (44%) to global TB cases. Thailand is in the top 30 countries for drug-resistant (DR) TB incidence (*1*). DR TB, including rifampin-resistant TB and strains with additional resistance to isoniazid (multidrug-resistant [MDR] TB), remains a great challenge for TB control. In 2018, ≈500,000 new cases of rifampin-resistant TB were reported globally, of which 78% were MDR TB (*1*). More worrisome is extensively drug-resistant (XDR) TB, which further exhibits resistance to 1 fluoroquinolone and 1 injectable second-line drug. The average proportion of global MDR TB cases with XDR TB is 6.2% (*1*). In Thailand, despite the reducing incidence of TB, the reported number of MDR TB cases nearly doubled during 2014–2018 (*1*); some are likely to be XDR TB. Treatment for patients with DR TB is prolonged and expensive, and outcomes are poor.

Whole-genome sequencing (WGS) of *M. tuberculosis* provides insights into drug resistance, in which mechanisms almost exclusively involve mutations (mostly single-nucleotide polymorphisms [SNPs], but also insertion/deletions) in genes coding for drug targets or drug-converting enzymes. WGS data can also provide insights into transmission and the dating of clusters (*2*), in which strains with near-identical genetic variants are likely to be part of a transmission chain (*3*). Analysis of *M. tuberculosis* WGS data from isolates across Thailand could provide much-needed insights into MDR/XDR TB transmission. Previous studies of DR TB have used genotyping techniques (e.g., spoligotyping, mycobacterial interspersed repetitive unit–variable-number tandem-repeat, and restriction fragment length polymorphism) (*4**,**5*), but these methods have limited resolution for inferring transmission because they investigate <1% of the *M. tuberculosis* genome. A recent WGS analysis revealed possible clonal transmission of 4 MDR TB isolates in Kanchanaburi Province (*6*). However, the extent of MDR TB and XDR TB clusters across Thailand is unknown. Our aim was to investigate the clustering patterns and risk factors of possible MDR TB, pre-XDR TB, and XDR TB transmission clusters across Thailand using WGS data.

## Methods

### Study Population and Setting

During 2014–2017, a total of 2,071 *M. tuberculosis* culture-confirmed MDR TB, pre-XDR TB, and XDR TB cases were listed in the laboratory records of the National Tuberculosis Reference Laboratory (NTRL; Ministry of Public Health) and Siriraj Hospital, Mahidol University, Thailand. These 2 laboratories cover 230 hospitals handling most DR TB cases in Thailand (Appendix 1 Tables 1, 2) (*7*). We randomly selected 547 *M. tuberculosis* isolates from MDR TB and pre-XDR TB cases across 6 regions and 71 of 77 provinces nationally. We also included all retrievable (n = 32) XDR TB isolates (Appendix 1 Table 3). For 11 cases, were used pairs of isolates collected at different times as internal controls for SNP distances. In each control pair, we included the isolate with the most mutations associated with drug resistance or the chronologically earlier isolate in the studied population (n = 579). We retrieved demographic data from laboratory records. The study protocol was approved by the Center for Ethics in Human Research, Khon Kaen University (approval no. HE601249).

### Phenotypic Drug-Susceptibility Testing

We performed phenotypic drug-susceptibility testing (DST) using the standard agar proportional method in Lowenstein-Jensen medium (*8*). Drug concentrations used were 0.2 μg/mL for isoniazid; 40.0 μg/mL for rifampin, ethionamide, capreomycin, and cycloserine; 2.0 μg/mL for ethambutol, ofloxacin, and levofloxacin; 4.0 μg/mL for streptomycin; 30.0 μg/mL for kanamycin; and 0.5 μg/mL for *para*-aminosalicylic acid. We used *M. tuberculosis* H37Rv as the susceptible reference strain.

### Whole-Genome Sequence Analysis

We used multiple loops of *M. tuberculosis* colonies for genomic DNA extraction (with the cetyl-trimethyl-ammonium bromide-sodium chloride method) (*9*). WGS data for 590 *M. tuberculosis* isolates were produced by NovogeneAIT (https://en.novogene.com) using the HiSeq (Illumina, https://www.illumina.com) platform generating 150-bp paired-end reads. We checked the quality of sequence reads using FastQC version 0.11.7 (*10*). We mapped high-quality reads from each isolate onto the H37Rv reference genome (GenBank accession no. NC_000962.3) using BWA-MEM version 0.7.12 (Li H, unpub. data, https://arxiv.org/abs/1303.3997). The average depth of sequencing coverage was high (341.01 ± 61.98). We used SAMtools version 0.1.19 (*11*) and GATK version 3.4.0 (*12*) to call SNPs and insertion/deletions. We filtered variants on the basis of a minimum coverage depth of 10-fold and Q20 minimum base-call quality score, and the intersection set of GATK and SAMtools variants was retained. We used the online tool TB-Profiler version 2.8.6 (*13**,**14*) to infer drug resistance and *M. tuberculosis* lineage membership on the basis of SNPs from the WGS data. The WGS data are available in the ENA Sequence Read Archive (https://www.ebi.ac.uk/ena/browser/home) (accession nos. PRJNA598981 and PRJNA613706).

### Phylogenetic Analysis

We constructed a phylogenetic tree based on 26,541 high-confidence SNPs among 590 isolates using the maximum-likelihood method with the selected general time-reversible with gamma-distribution model, implemented within MEGA version 10.1 (*15*). We excluded the 130 SNPs known to be associated with DR TB found in this study to ensure that they would not affect the phylogenetic analysis. We inferred a bootstrap consensus tree from 1,000 replicates. We produced the phylogenetic tree image using iTOL (*16*).

### Data Analysis

Isolates forming monophyletic groups in which many or all pairs differed by <25 SNPs were placed in the same clade. Clusters included isolates differing by 0–11 SNPs. We regarded members of a single cluster as possibly descended from a single clone through recent transmission. Less-recently transmitted isolates within a clade differed by 12–25 SNPs. We calculated the clustering percentage as (no. clustering isolates/total no. isolates) × 100. We differentiated isolates with acquired DR TB from possible primary DR TB (MDR TB, pre-XDR TB, and XDR TB) isolates on the basis of acquisition of additional resistance-associated mutations, especially those associated with resistance to fluoroquinolones, kanamycin, or capreomycin, drugs that are used for DR TB classification. For clusters containing isolates with different types of DR TB (such as MDR TB and XDR TB), we used the acquisition of additional drug-resistance SNPs and co-ancestral relationships to differentiate between 2 patterns of acquired resistance: chronological (ancestral strain had fewer mutations, lesser type of DR, or both) or nonchronological (ancestral strain had more mutations, stronger type of DR, or both). Although XDR TB and pre-XDR TB could be considered as subsets of MDR TB, we have treated all 3 as separate categories in our analyses.

We analyzed all data using R statistical software version 3.6.1 (https://www.r-project.org) and considered p values <0.05 to be statistically significant. We analyzed associations between clades/clusters and geography using χ^2^ tests and visualized them with the R package vcd version 1.4–8. We calculated odds ratios (ORs) with 95% CIs using the R package epiR version 1.0–4. We tested factors associated with clustering isolates using the Student *t*-test (numerical data), χ^2^ test, or Fisher exact test (categorical data), when applicable. We constructed graphs using the R package ggplot2 version 3.2.1 and built phylo-maps using the package phytools version 0.7–20.

## Results

### Study Population and Characteristics

**Figure 1:**
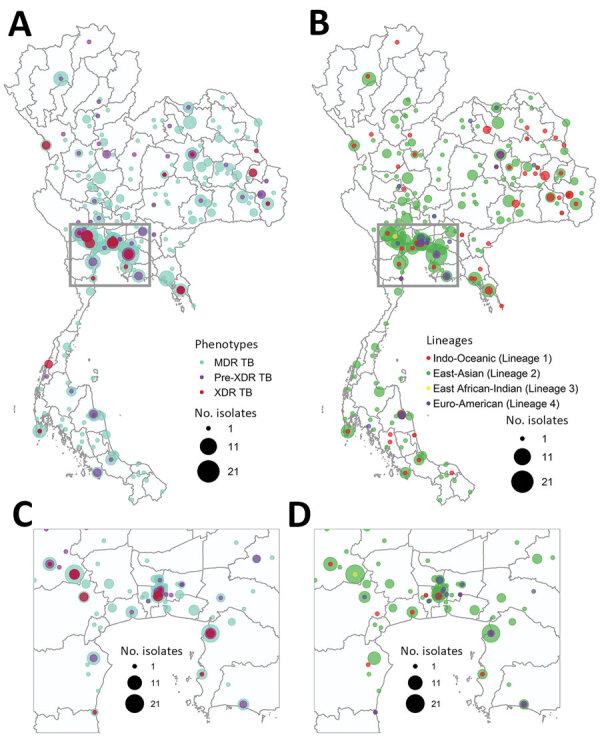
Geographic and lineage distribution of 579 drug-resistant *Mycobacterium tuberculosis* isolates in Thailand, 2014–2017. A) Geographic distribution of MDR TB, pre-XDR TB, and XDR TB. B) Lineage distribution of drug-resistant *M. tuberculosis*. C) Drug-resistant types, enlarged from panel A. D) Lineage distribution, enlarged from panel B. The size of each circle is proportional to the number of isolates. MDR, multidrug resistant; TB, tuberculosis; XDR, extensively drug-resistant.

Most (466; 80.5%) of the 579 culture-confirmed DR TB cases in the studied population were MDR TB, followed by 81 pre-XDR TB (14.0%) (Appendix 1 Table 2). We included all available XDR TB isolates (n = 32), constituting 5.5% of our samples but only 1.5% of the culture-confirmed 2,071 DR TB isolates collected nationally during 2014–2017. Central and northeast regions of Thailand had the highest DR TB proportions (Figure 1). The 3 provinces with the highest number of DR TB cases were Bangkok (n = 85; 14.7%), Kanchanaburi (n = 51; 8.8%), and Chonburi (n = 37; 6.4%) (Figure 1; Appendix 1 Table 3). Most patients were male (n = 419; 73.1%) and mean age was 43.5 (±14.7) years (Appendix 1 Table 4).

### Phylogenetic Analysis

**Figure 2:**
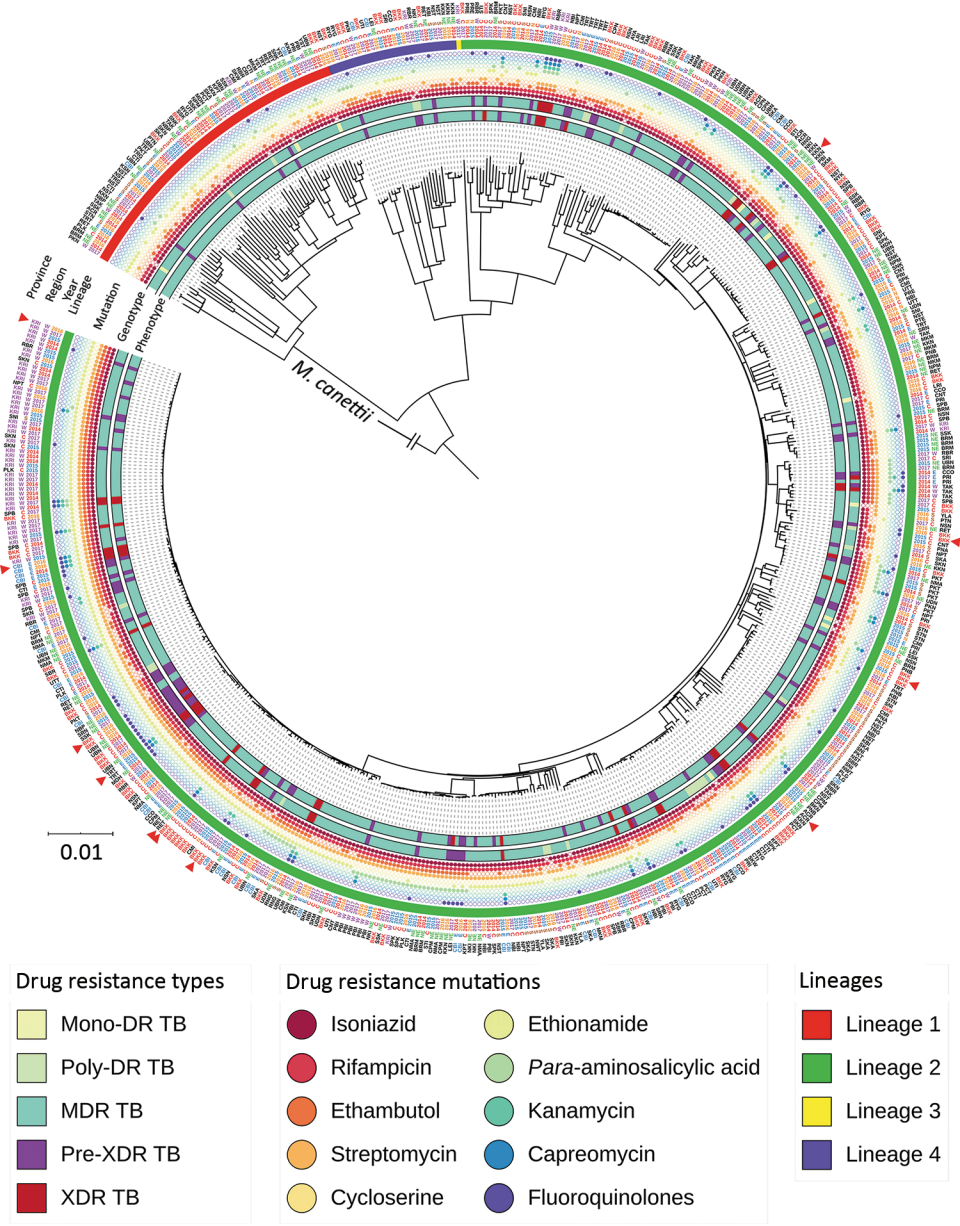
Phylogenetic tree for the 590 drug-resistant *Mycobaterium tuberculosis* isolates from Thailand, 2014–2017. From inner to the outer circles: culture-based phenotypic drug-susceptibility test, whole-genome sequencing–based drug-resistance profile (DR TB, MDR TB, pre-XDR TB, and XDR TB), drug-resistance mutations, lineage, year of collection, regions, and provinces. Red triangles indicate the paired isolates from the same patients (n = 11). Scale bar indicates the genetic distance proportional to the total number of single nucleotide polymorphisms. *M. canetti* was used as an outgroup. DR TB, drug-resistant tuberculosis; MDR, multidrug resistant; TB, tuberculosis; XDR, extensively drug-resistant.

Most of the *M. tuberculosis* isolates belonged to the East-Asian lineage (lineage 2) (n = 482; 83.2%), followed by the Indo-Oceanic lineage (lineage 1) (n = 67; 11.6%), the Euro-American lineage (lineage 4) (n = 29; 5.0%), and the East African-Indian lineage (lineage 3) (n = 1; 0.2%) (Figure 2; Appendix 1 Table 5). Lineage 2.2.1 (n = 413; 71.3%) was the main sublineage among MDR, pre-XDR, and XDR TB.

### Clustering and Possible Transmission Clusters

**Figure 3:**
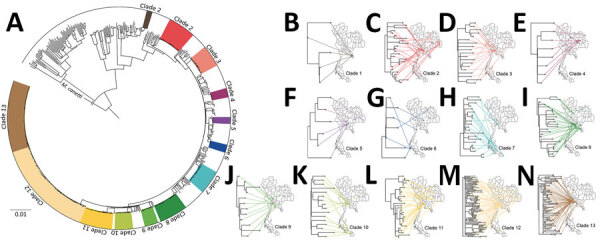
Geographic distribution of 13 major clades of drug-resistant tuberculosis in Thailand. A) The 13 clades are highlighted in the outer circle. Scale bar indicates the genetic distance proportional to the total number of single nucleotide polymorphisms. B–N) Each of the 13 major clades is associated with particular geographic regions, as shown. *Mycobacterium canetti* was used as an outgroup.

The phylogenetic tree (Figure 2) showed enormous diversity among the DR TB isolates from Thailand. Many isolates were distinct, differing from all others at a mean ±SD of 657 ± 626 SNPs. Most isolates (n = 319; 55.1%) grouped into 13 clades, each consisting of 5–86 isolates (Figure 3; Appendix 2 Figure 1). Clades 1, 6, 11, and 13 each consisted of a single small cluster of closely related isolates; the remaining clades included >1 possible clusters (Appendix 2 Figure 2). The isolates grouped in each clade were significantly associated with a particular geographic region (p<0.001; Appendix 2 Figure 3, panel A). Clade 1 (Figure 3, panel B) was found only in Trat Province and clade 13 predominated in Kanchanaburi (Figure 3, panel N).

**Figure 4:**
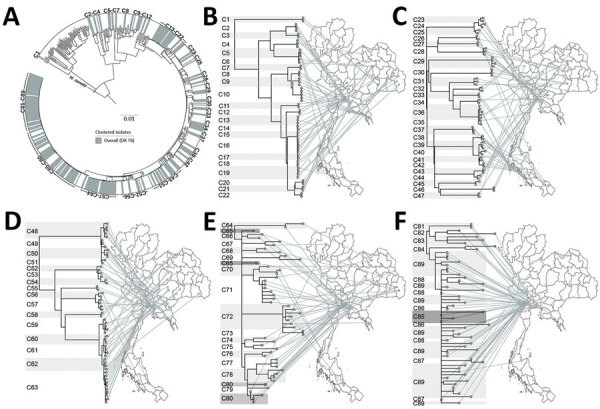
All clusters of DR TB isolates from Thailand. A) A total of 89 clusters are highlighted in the outer circle. Scale bar indicates the genetic distance proportional to the total number of single nucleotide polymorphisms. B–F) Phylogeographical links of each cluster are shown. For clarity, clusters are divided among 5 phylomaps. Some isolates in closely related clusters (C64–C65, C79–C80, and C85–C89) crossed localities. *Mycobacterium canetti* was used as an outgroup. DR TB, drug-resistant tuberculosis.

A total of 89 clusters contained 281 isolates (48.5%) (Appendix 1 Table 6). Sixty clusters (isolates differing by ≤11 SNPs), containing 2–34 isolates, fell within the major clades. A further 29 smaller clusters occurred elsewhere in the tree. Most isolates within a cluster shared geographic links (Figure 4, panels A–F; Appendix 1 Table 6). The percentages of MDR TB, pre-XDR TB, and XDR TB isolates (based on phenotypic DST) that fell into clusters were 46.1% (215/466) for MDR TB, 49.4% (40/81) for pre-XDR TB, and 81.3% (26/32) for XDR TB (Appendix 1 Table 6). Pairwise SNP distances within and between each of the 89 clusters are given summarized (Appendix 1 Table 7).

Some clusters included isolates with different types of DR TB. Nineteen of the 89 clusters (C2, C7, C10, C16, C22, C36, C37, C40, C43, C49, C59, C60, C63, C70, C72, C76, C80, C83, and C89) had a chronological pattern based on the progressive increase in numbers of DR mutations from base to tips in the phylogeny (Appendix 1 Table 8). The pattern of DR mutation changes was nonchronological in clusters C21, C23, C32, C35, C41, C55, C71, and C75. Among the 281 clustering isolates, 81.9% were classified as possible primary DR TB (n = 230), including MDR TB (n = 176/205; 85.9%), pre-XDR TB (n = 29/46; 63.0%), and XDR TB (n = 14/19; 73.7%). In addition, we identified 10 phenotypically MDR isolates and 1 phenotypically pre-XDR TB isolate as possible examples of primary isoniazid resistance (n = 11) based on genotypic DR. Other clustering isolates (n = 51/281, 18.1%) exhibited acquired DR TB (MDR TB [n = 29/205; 14.1%], pre-XDR TB [n = 17/46; 37.0%], and XDR TB [n = 5/19; 26.3%]) (Table 1).

**Table 1:** Characteristics of isolates within 89 DR TB clusters, Thailand, 2014–2017*

Clustered isolates, n = 281	DR TB types, no. (%)†			
	INH-R, n = 11	MDR TB, n = 205	Pre-XDR TB, n = 46	XDR TB, n = 19
Possible primary DR TB,‡ n = 230, 81.85%	11 (100.0)	176 (85.85)	29 (63.04)	14 (73.68)
Possible acquired DR TB,‡ n = 51, 18.15%	0	29 (14.15)	17 (36.96)	5 (26.32)

*Using a pairwise-difference range of 0–11 single nucleotide polymorphisms, 89 clusters (minimum cluster size = 2 isolates) were recognized. DR, drug-resistant; INH-R, isoniazid resistant; MDR, multidrug-resistant; TB, tuberculosis; XDR, extensively drug-resistant. †DR TB types based on genotypic drug susceptibility tests. ‡Possible primary DR TB isolates were differentiated from acquired DR TB isolates based on the acquisition of mutations associated with DR TB and from co-ancestral relationships.

Among clustered isolates, there was some discordance between phenotypic DST findings (MDR TB [n = 215], pre-XDR TB [n = 40], and XDR TB [n = 26]) and genotypic DST results (poly-DR TB [n = 11], MDR TB [n = 205], pre-XDR TB [n = 46], and XDR TB [n = 19]) (Appendix 1 Table 8). We identified 11 isolates of phenotypically MDR TB genotypically as poly-DR TB (resistant to >1 drug but not to both isoniazid and rifampicin). We identified 66 MDR TB, 9 pre-XDR TB, and 10 XDR TB clusters on the basis of phenotypic DST (Appendix 1 Table 9; Appendix 2 Figure 4, panels A–F). Most pre-XDR TB and XDR TB clusters had hospital-based links (Appendix 1 Table 9). All phenotypic DR TB clusters and resistance types, stratified by province, are shown (Appendix 1 Table 10).

### Factors Associated with Possible DR TB Transmission Clusters

TB patients from whom clustering isolates were obtained had an average age of ≈42 years. Isolates falling within clusters were significantly associated with geographic regions (p = 0.001; Appendix 2 Figure 3, panel B). Patients with TB who lived in western provinces had a higher risk of being within possible DR TB transmission clusters than those elsewhere (OR 2.44, 95% CI 1.53–3.89; Table 2). Lineage 2.2.1 (versus other lineages) was associated with a higher risk of possible DR TB transmission clusters (OR 3.59, 95% CI 2.42–5.32). Lineage 1 had the lowest risk of being represented in DR TB transmission clusters (OR 0.03, 95% CI 0.01–0.11). Clustering isolates had drug-resistance mutations such as *katG* S315T, *rpoB* S450L, and *embB* G406D (Table 2).

**Table 2:** Demographic and other factors associated with clustering (≤11 SNP difference) of TB isolates, Thailand, 2014–2017*

Characteristic	All isolates, n = 579	Clustering isolates		Odds ratio (95% CI)
		Isolates falling within clusters, n = 281	Nonclustering isolates, n = 298	
Sex, n = 573				
M	419 (73.12)	198 (70.71)	221 (75.43)	0.79 (0.54–1.14)
F	154 (26.88)	82 (29.29)	72 (24.57)	1.27 (0.88–1.84)
Age, y, n = 508				
Mean ± SD	43.51 ± 14.68	42.02 ± 15.23	44.94 ± 14.03	NA

Region				
Central	183 (31.61)	79 (28.11)	104 (34.90)	0.73 (0.51–1.04)
Eastern	88 (15.20)	47 (16.73)	41 (13.76)	1.26 (0.80–1.98)
Northeastern	125 (21.59)	56 (19.93)	69 (23.15)	0.83 (0.56–1.23)
Northern	17 (2.94)	4 (1.42)	13 (4.36)	0.32 (0.10–0.98)
Southern	73 (12.61)	33 (11.74)	40 (13.42)	0.86 (0.52–1.40)
Western	93 (16.06)	62 (22.06)	31 (10.40)	**2.44 (1.53–3.89)**

Lineage				
2.1	31 (5.35)	12 (4.27)	19 (6.38)	0.66 (0.31–1.38)
2.2.1	413 (71.33)	236 (83.99)	177 (59.40)	**3.59 (2.42–5.32)**
2.2.1.1	32 (5.53)	16 (5.69)	16 (5.37)	1.06 (0.52–2.17)
2.2.1.2 and 2.2.2	6 (1.04)	2 (0.71)	4 (1.34)	0.53 (0.05–3.71)
4	29 (5.01)	13 (4.64)	16 (5.35)	0.86 (0.41–1.82)
1	67 (11.57)	2 (0.71)	65 (21.81)	**0.03 (0.01–0.11)**
3	1 (0.17)	0 (0.00)	1 (0.34)	NA

Drug-resistance mutations				
Isoniazid, n = 565				
*katG* S315T	448 (79.29)	252 (89.68)	196 (69.01)	**3.90 (2.46–6.18)**
*inhA* −15c/t	52 (9.20)	7 (2.49)	45 (15.85)	**0.14 (0.06–0.31)**
Rifampin, n = 554				
*rpoB* S450L	279 (50.36)	176 (65.19)	103 (36.27)	**3.29 (2.32–4.66)**
Ethambutol, n = 335				
*embB* M306V	85 (25.37)	44 (20.75)	41 (33.33)	**0.52 (0.32–0.86)**
*embB* G406D	66 (19.70)	59 (27.83)	7 (5.69)	**6.39 (2.81–14.51)**
*embB* M306I	56 (16.72)	27 (12.74)	29 (23.58)	**0.47 (0.26–0.84)**
Streptomycin, n = 349				
*rpsL* K43R	295 (84.53)	188 (89.95)	107 (76.43)	**2.76 (1.52–5.01)**
Ethionamide, n = 268				
*ethA* 639–640del	143 (53.36)	105 (73.43)	38 (30.40)	**6.33 (3.72–10.77)**
*inhA* −15c/t	65 (24.25)	9 (6.29)	56 (44.80)	**0.08 (0.04–0.18)**
*Para*-aminosalicylic acid, n = 99				
*folC* S150G	39 (39.39)	32 (50.79)	7 (19.44)	**4.28 (1.63–11.19)**

*Values are no. (%) except as indicated. Bold type indicates statistical significance. NA, not applicable.

## Discussion

MDR TB and XDR TB are serious global problems, but few studies have focused on their transmission at a nationwide resolution. Thailand has a high burden of MDR TB and increasing numbers of MDR TB cases (*1*). We sourced 579 DR TB isolates across 71 provinces during 2014–2017. Nearly half of these were in possible transmission clusters, mostly involving *M. tuberculosis* lineage 2.2.1. A total of 89 clusters, most distributed among 13 major clades, contributed to multiclonal MDR TB outbreaks associated with specific regions in Thailand. Bangkok, Kanchanaburi, and Chonburi were the provinces with the highest proportions of MDR TB, pre-XDR TB, and XDR TB clusters (i.e., groups of isolates differing by <11 SNPs). We used 2 criteria to select SNP cutoff values. First, the <11 SNP difference cutoff for a cluster was derived directly from the maximum number of differences between the 11 paired isolates used as an internal control. Second, we used an SNP cutoff concordant with, or more stringent than, those in previous studies (*17**–**20*). Our 11-SNP cutoff was proportionally 0.0004 of the 26,541 SNPs in our total set. This proportion was concordant with that in a previous study (*21*), and more stringent than those in other studies (*18**,**20*). A <12-SNP cutoff has been previously proposed as the upper boundary for possible cluster transmission events (*2*).

Phylogenetic analysis identified 13 major clades, each associated with a particular region(s). Pairwise SNP differences between isolates within clades ranged from <11 to ≈25, suggesting a range of divergence times from a common ancestor (Appendix 2 Figure 2). On the basis of the transmission time estimates (0.5 SNP/genome/year) for *M. tuberculosis* (*2*), some of these major clades might have begun to circulate in Thailand ≈20–40 years ago, others more recently. Isolates differing by 12–25 SNPs nevertheless often shared geographic links. For example, 17 of 21 (81%) isolates in clade 7 (Figure 3, panel H), which had pairwise differences indicating a relatively nonrecent common ancestor, were located within neighboring provinces of southern Thailand. Clades 1, 6, 11, and 13 each consisted of isolates differing at very few SNPs, giving us confidence that these were likely examples of recent transmission. Nonetheless, isolates in clade 6 often occurred in different provinces.

The largest and most recent clade was clade 13 (Figure 3, panel N), comprising 62 cases (46 MDR TB, 11 pre-XDR TB, and 5 XDR TB based on phenotypic DST) found in the western region, especially in Kanchanaburi. This finding suggests that clones of pre-XDR TB and XDR TB may emerge from recent MDR TB ancestors. We confirmed a previous report (*22*) that there was a large MDR TB outbreak in Kanchanaburi. In addition, clade 13 is sister to clade 12, which consists of strains that spread in both central (especially Bangkok) and northeast Thailand and contain less recently transmitted strains. Therefore, the MDR TB outbreak clade in Kanchanaburi was derived from a less recently transmitted clade elsewhere in Thailand.

We identified 89 clusters (isolates in each differing by <11 SNPs) of DR TB in Thailand. The clustered isolates showed a strong association with geographic region. The largest cluster (C89), within clade 13 in Kanchanaburi, comprised 34 isolates (27 MDR TB and 7 pre-XDR TB based on phenotypic DST). In South Africa, WGS analysis of a large XDR TB cohort (>400 cases) from a single province showed that only 30% of participants had clear epidemiologic links (person–person or hospital link): 70% of transmission events may have resulted from casual contact between persons not known to one another (*23*). Another study in South Africa showed that 19% of XDR TB patients discharged from the hospital caused secondary XDR TB cases in the community (*24*). Here, we found 9 clusters of pre-XDR TB (the largest with 7 isolates) and 10 clusters (the largest with 4 isolates) of XDR TB in Thailand (Appendix 1 Table 9; Appendix 2 Figure 4).

To reflect the extent of the DR TB outbreak in Thailand, we calculated the proportion of isolates falling into the 89 DR TB clusters (Table 1). In some clusters, isolates exhibited different types of DR TB associated with chronology, revealing the progression of DR mutations in the phylogeny, moving from the ancestor toward the tips of the tree (Appendix 1 Table 8). Based on mutation-acquisition analysis within this phylogeny, we saw examples of possible primary resistance in 85.9% of MDR TB, 63.0% of pre-XDR TB, and 73.7% of XDR TB cases. Eight clusters included isolates with different types of DR and more resistance-associated mutations in the ancestral strain than in its descendants. This situation might be explained by different durations of the latency stage occurring after transmission events leading to the emergence of less troublesome DR TB cases (such as MDR TB) later than the more troublesome cases (such as XDR TB) (*25*). Because not all cases from the possible transmission chain could be included, undetected primary resistance might exist. Data from all DR TB cases in the community and information on treatment history and known exposure are needed to accurately and completely estimate the extent of primary DR TB. The proportion of primary DR TB cases could be higher because we reported numbers of MDR TB cases excluding pre-XDR TB and XDR TB (each of which was reported as a separate subset). In addition, some index cases might not have been included in the selected population.

Previously reported factors contributing to MDR TB transmission include illicit drug usage (*26*); delayed TB diagnosis and being >45 years of age (*18*); and being single, having low income, suffering frequent stress and other diseases, and lacking medical insurance (*27*). Lineage 2 predominated in previous studies of transmission of MDR TB (*18**,**26**,**28*). We found that infection with lineage 2.2.1 is the strongest predictor (3.6-fold) of DR TB clusters, whereas infection with lineage 1 had the lowest risk. Living in the western region of Thailand increased the risk of being in DR TB clusters by 2.4-fold. The western region, being close to the border with Myanmar, differs from other regions of the country in terms of both ethnicity and economic development. These differences might explain the increased risk there (*29*). Previously, clustering isolates were more likely to have mutations of *rpoB* S450L (*18**,**30*), *katG* S315T, or the *inhA* promoter (*31*). We also found a pattern of drug resistance-associated mutations (*katG* S315T, *rpoB* S450L, *embB* G406D, *rpsL* K43R, *ethA* 639–640del, and *folC* S150G) in clusters.

The DR TB situation in Thailand is a major concern and requires urgent implementation of control measures such as active case finding to disrupt the transmission chain and targeted intervention and contact tracing in hotspot regions. The mortality rate and cost of treatment of XDR TB is very high (*32*); therefore, these DR types should be the priority for intervention. The large size of some clusters might reflect their high transmissibility (*33*); thus, tracking clade 13 at Kanchanaburi should be a priority. Besides the 13 major clades, several small clusters of DR TB were found in many provinces. The potential for expansion of these small clusters is unknown. Here, we also identified the hotspot provinces to help prioritize locations for intervention.

Globally, few studies at the nationwide scale have used WGS analysis of MDR TB, pre-XDR TB, and XDR TB (*26**,**30**,**34**–**36*). Older studies have used blunt genotyping tools (e.g., IS*6110* restriction fragment length polymorphism, spoligotyping, and mycobacterial interspersed repetitive unit–variable-number tandem-repeat) with limited or convenient sample sizes. DR TB studies using WGS in Saudi Arabia and Portugal have revealed transmission clusters of MDR TB; however, they had small samples and provided limited data on epidemiologic links (*36**,**37*). Extrapolating from our findings, primary-resistant TB strains may be the main contributors to the current global problem of high MDR TB and XDR TB prevalence.

The primary limitations of our study were that it was retrospective rather than prospective, lacked socioeconomic data for analysis, and lacked fine-scale data of epidemiologic links: possible transmission clusters were presumed only from the genetic distances among isolates and each patient’s hospital and province of residence. In addition, an accurate estimation of the exact time of the possible transmission cannot be made: clusters originating years ago may be continuing to spread. We also lacked information about treatment and exposure history and of the complete population to identify all index cases to differentiate between primary and acquired DR TB. In addition, the prevalence and clustering of MDR TB, pre-XDR TB, and XDR TB isolates in some provinces might be underestimated because of the low coverage of DST for the first-line drugs among TB cases (*1*).

In conclusion, we have demonstrated the usefulness of WGS for DR TB epidemiology. We have shown that close to half of MDR TB, pre-XDR TB, and XDR TB cases in Thailand might be caused by transmission clusters. Two thirds of pre-XDR TB and three quarters of MDR TB and XDR TB clustering isolates were possible examples of primary resistance. These results indicate that the emergence of MDR TB, pre-XDR TB, and XDR TB cases in Thailand might be from a narrow base of ancestral strains. The high prevalence of MDR/XDR TB in Thailand might be the result of multiclonal outbreaks. People living in the western region of Thailand had a 2.4-fold increased risk of DR TB clusters, and lineage 2.2.1 conferred a 3.6-fold increased risk of forming DR TB clusters relative to other lineages.

Appendix 1 Additional tables providing information on clusters of drug-resistant *Mycobacterium tuberculosis*, Thailand, 2014–2017.

Appendix 2 Additional figures describing clusters of drug-resistant *Mycobacterium tuberculosis*, Thailand, 2014–2017.
