# Supplementary material for: Clusters of Drug-Resistant Mycobacterium tuberculosis Detected by Whole-Genome Sequence Analysis of Nationwide Sample, Thailand, 2014–2017
Source: Emerg Infect Dis. 2021 Mar;27(3):813–22. doi: 10.3201/eid2703.204364 (PMC7920678; doi:10.3201/eid2703.204364)
Supplement: Appendix 2 — Additional figures describing clusters of drug-resistant Mycobacterium tuberculosis, Thailand, 2014–2017. [file 20-4364-Techapp-s2.pdf]

Article DOI: https://doi.org/10.3201/eid2703.204364

Clusters of Drug-Resistant *Mycobacterium tuberculosis* Detected by Whole-Genome Sequence Analysis of Nationwide Sample, Thailand, 2014–2017

Appendix 2

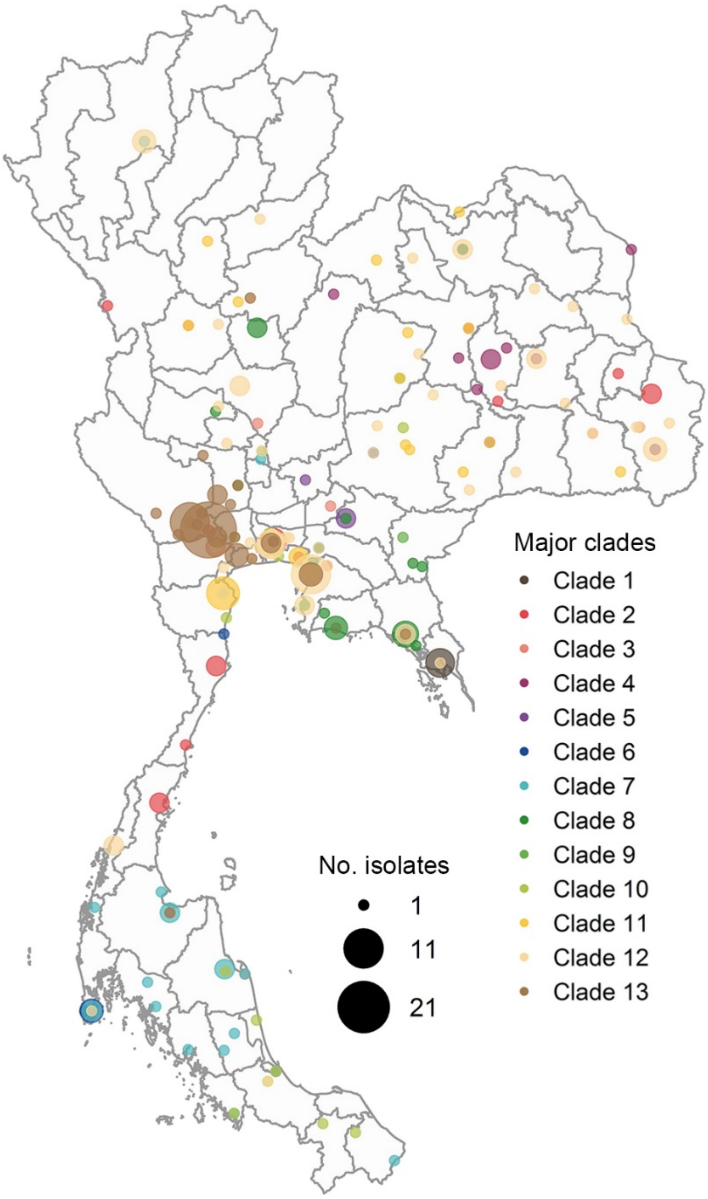

Appendix 2 Figure 1. Geographical distribution of 319 isolates from 13 major clades across Thailand. The size of each circle is proportional to the number of isolates.

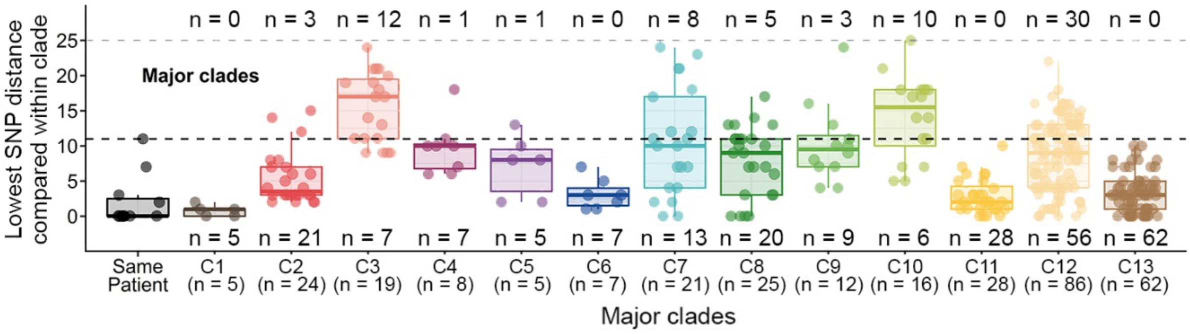

Appendix 2 Figure 2. Comparisons of the proportion of isolates in each clade that differ by ≤11 single nucleotide polymorphisms (SNPs) (suggesting recent transmission) and those that differ by 12–25 SNPs in many or all pairs (suggesting less-recent transmission).

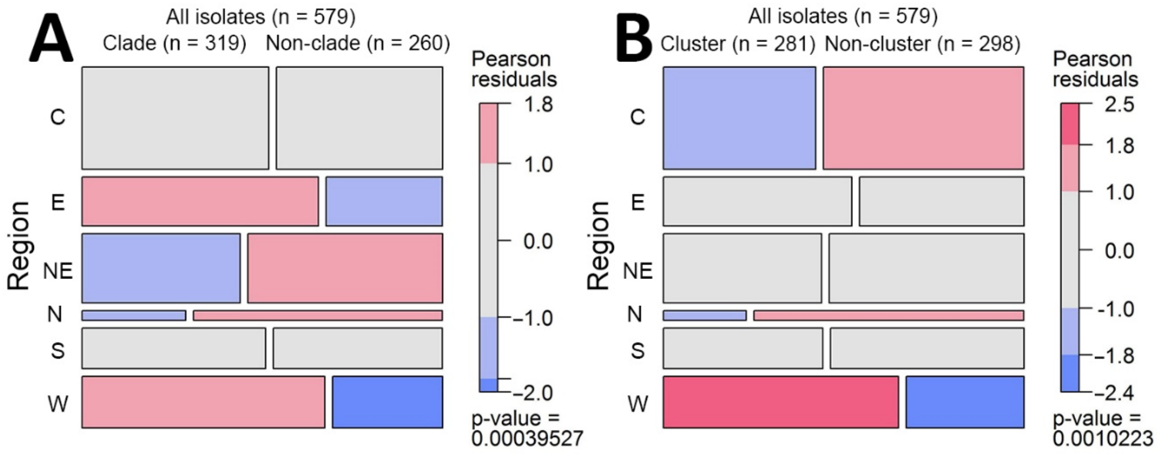

Appendix 2 Figure 3. Association between geographical regions and A) 13 clades; B) 89 clusters.

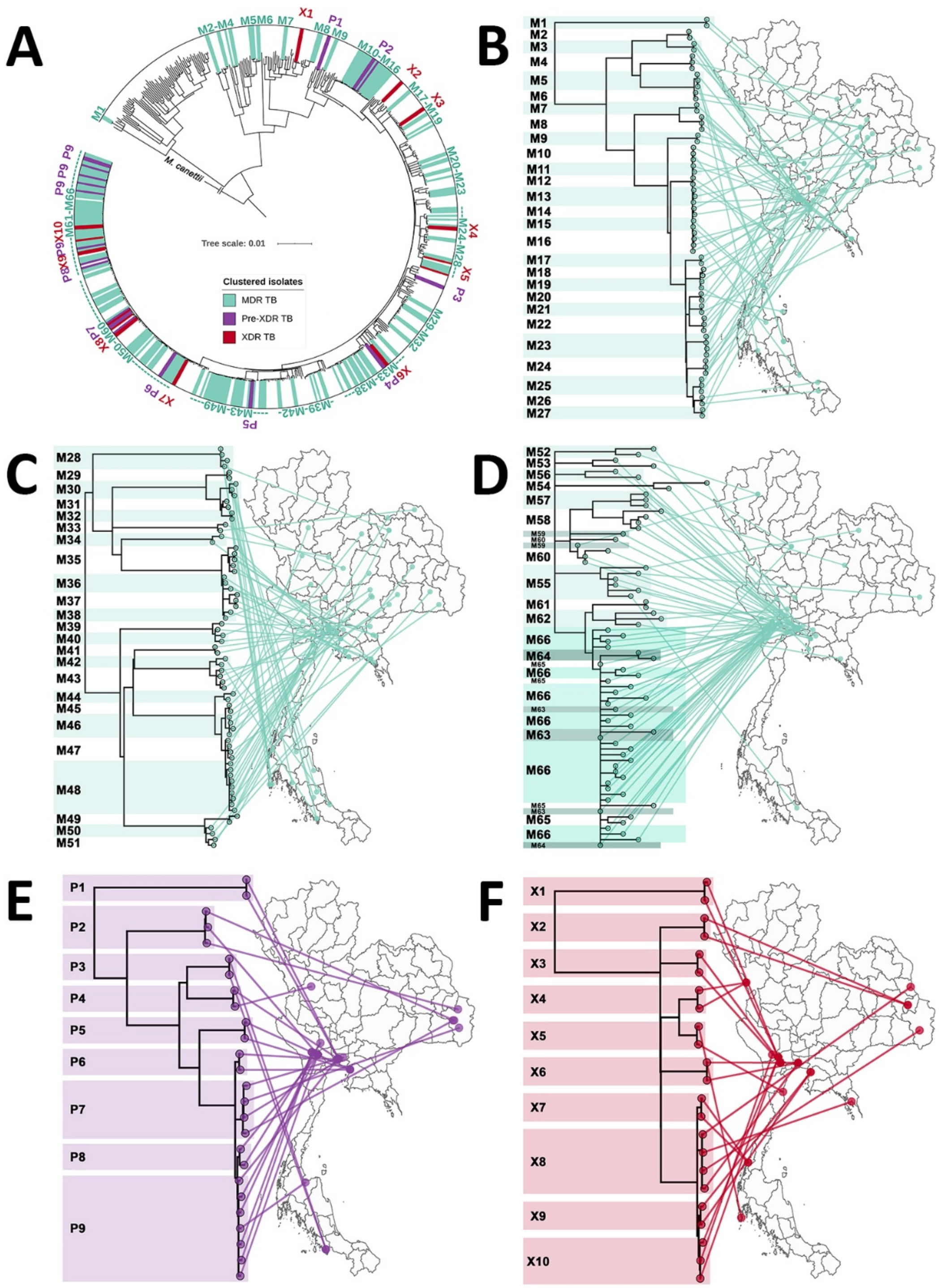

Appendix 2 Figure 4. Clusters of multidrug-resistant tuberculosis (MDR TB), pre-extensively drug-resistant tuberculosis (pre-XDR TB), and extensively drug-resistant tuberculosis (XDR TB) isolates based on phenotypic drug-susceptibility test. A) 66 MDR TB (M1–M66), 9 pre-XDR TB (P1–P9), and 10 XDR TB (X1–X10) clusters are highlighted in the outer circle. Phylogeographical links of MDR TB (B–D), pre-XDR TB (E), and XDR TB (F) clusters are shown. Some isolates in closely related clusters (M59–M60 and M63–M65) crossed localities. For clarity, MDR TB clusters are split among 3 phylomaps (B–D).
